# Microencapsulation by a Spray Drying Approach to Produce Innovative Probiotics-Based Products Extending the Shelf-Life in Non-Refrigerated Conditions

**DOI:** 10.3390/molecules28020860

**Published:** 2023-01-15

**Authors:** Giuseppina Gullifa, Roberta Risoluti, Cristina Mazzoni, Laura Barone, Elena Papa, Alfredo Battistini, Rodrigo Martin Fraguas, Stefano Materazzi

**Affiliations:** 1 Department of Chemistry, “Sapienza” University of Rome, p.le A.Moro 5, 00185 Rome, Italy; 2 Department of Biology and Biotechnologies “C. Darwin”, Sapienza University of Rome, Piazzale Aldo Moro 5, 00185 Roma, Italy; 3 Consiglio per la Ricerca in Agricoltura e l’Analisi dell’economia Agraria—Centro di Politiche e Bioeconomia, Via Pò 14, 00198 Rome, Italy; 4 Department of Chemistry, Campus Varginha, University of José do Rosário Vellano—UNIFENAS, Alfenas 37130-000, Brazil

**Keywords:** microencapsulation, spray drying, probiotics, nutraceutics

## Abstract

Recently, there has been a growing interest in producing functional foods containing encapsulated probiotic bacteria due to their positive effects on human health. According to their perceived health benefits, probiotics have been incorporated into a range of dairy products, but the current major challenge is to market new, multicomponent probiotic foods and supplements. Nevertheless, only a few products containing encapsulated probiotic cells can be found as non-refrigerated products. In this work, spray drying technology was investigated in order to produce an innovative nutraceutical formulation based on lactic acid bacteria (LAB), and was able to ensure a good storage stability of probiotics (no less than 109 CFU/cps) in non-refrigerated conditions. Probiotic-loaded microparticles from spray drying experiments were produced under different conditions and compared by thermogravimetric analysis (TGA), scanning electron microscopy (SEM), and the enumeration of the number of viable cells in order to identify the formulation exhibiting the most promising characteristics. Results from the dissolution test revealed that the optimized formulation provides a suitable amount of living cells after digestion of microparticles stored for 12 months at room temperature and confirmed that the microencapsulation process by spray drying ensures a good protection of probiotics for nutraceutical purposes.

## 1. Introduction

The diversification of the market of probiotic foods relies on the availability of new strains or new formats of probiotic cultures [1].

Until now, fermented dairy products, mainly fermented milks, have been used as the most successful commercial food products for the delivery of probiotic bacteria, as frozen and freeze-dried cultures are the commercially available formats of starter and probiotic bacteria [2,3]. In particular, the production of dried cell cultures is particularly interesting because, unlike frozen cultures, dehydrated cultures demand less storage capacity and lower cost of transport and refrigeration [4]. However, the maintenance of cell viability during drying and storage is a major challenge [5,6,7].

The presence of different human digestive tract conditions makes it challenging to design a delivery system of probiotics, but also presents the opportunity to produce highly customized systems, so as to deliver the capsules to the desired location [8,9,10].

Therefore, the material used would be of relevance. These materials generally consist of a polymeric matrix; the addition or the mixing of polymers has the potential to give a modified release of microencapsulated probiotics [11].

The main features of microencapsulation regarding probiotics are that it provides a good protection against acids and that the preparation process is carried out gently, so as not to damage the trapped cells [12,13,14]. The polymer used must be non-cytotoxic, as well as non-antimicrobial. Furthermore, the material must be edible if used in the food industry (as in the case of probiotics), and be able to form a barrier to protect the encapsulated substance.

Gums are the most used materials for probiotic encapsulation (sodium alginate, xanthan gum, gum arabic and carageenan). In the literature to date, the most common encapsulation agent is represented by sodium alginate [15], a linear polysaccharide composed of the salt sodium alginic acid; it is particularly suitable for encapsulation of microorganisms because of the gelling conditions, the state of GRAS and for its lack of toxicity [16]. Other polysaccharides (such as chitosan, often used in combination with alginate) are widely used and a number of these show good potential as an encapsulation matrix for protecting the encapsulated bacteria from the harsh conditions of bile and stomach acid. Other materials commonly used are lipids (stearic acid and phospholipids) [17], cellulose (carboxymethylcellulose and methylcellulose), carbohydrates (starch and sucrose) and proteins (albumin and gelatin). In particular, among the materials considered in this study, Eudragit polymers are copolymers derived from esters of acrylic and methacrylic acid, whose physicochemical properties are determined by their functional groups (R). Eudragit polymers are available in a wide range of different physical forms (aqueous dispersion, organic solution granules and powders) [18,19,20].

Microencapsulation with a spray drying process is frequently used in the food industry to improve the availability of food supplements [21,22,23,24].

At its beginning, this process was applied to flavorings, in order to protect them from degradation/oxidation, and also to dry solid suspensions, but it is presently applied to bioactive molecules and probiotics. Spray drying is a relatively low-cost technology; it is rapid and reproducible, allowing easy scale-up, when compared to other microencapsulation techniques, justifying its use in industrial purposes [25,26,27,28,29].

Atomization can be performed by different types of atomizers (pneumatic atomizer, pressure nozzle, spinning disk, fluid nozzle and sonic nozzle). In addition, despite the high temperatures involved in the process, which may not always be suitable for the encapsulation of probiotic bacterial cultures, the advantage of the process is that it can be operated in a continuous manner [30,31].

In this work, an innovative analytical strategy based on spray drying technology was optimized to introduce probiotics into nutraceutical products claimed as food supplements. In order to evaluate the characteristics of the coated probiotics in an oral dosage form, thermogravimetric analysis (TGA) was used to estimate the effectiveness of the coating process, while scanning electron microscopy (SEM) was performed to investigate the morphology of the microparticles. In addition, the enumeration of the colony forming units per capsule (CFU/cps) after digestion was estimated by using simulated fluids. The optimal formulation provided a good storage stability (no less than 109 CFU/cps) in non-refrigerated conditions for a period of 12 months.

## 2. Results and Discussion

### 2.1. Coating Selection for Spray Drying Technology

Producing stable microspheres for microbial cell isolation starts with the selection of an appropriate encapsulation material. Several aqueous enteric coating systems suitable for spray drying applications in conventional dosage forms are now available in the market; among those allowed as food supplements, poly(meth)acrylate polymers were selected as pH-responsive coating agents, commonly available under the tradename Eudragit. The reason why these polymers were chosen as model materials for spray drying applications is related to the different ratio of monomers (two to three different methacrylate monomers, methacrylic acid, methacrylic acid esters and dimethylaminoethyl methacrylate), making them suitable matrices for targeted release/delivery systems in the GI tract.

In addition, methacrylate copolymers represent interesting candidates for the production of microparticles by spray drying since they are inert and freely soluble in organic and aqueous solvents [32].

Among the different types of commercialized Eudragit, L30 D55 type is a pH-dependent enteric polymer composed of a methacrylic acid-ethyl acrylate copolymer (1:1), soluble from a pH of 6.8.

This kind of Eudragit is insoluble in the mouth and stomach and it starts to dissolve in the duodenum (pH around 6). Since the pH in the colon is around pH 7.5, Eudragit L 30 D55 microparticles can be used for the delivery of active ingredients to the lower part of the intestine and also in the jejunum and ileum.

### 2.2. Thermal Analysis Results

**Figure 1:**
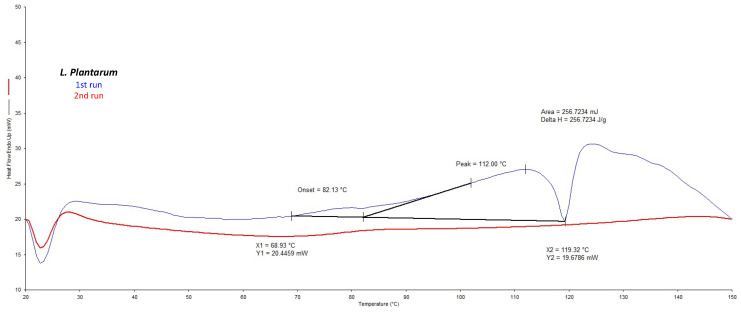
DSC curves of the first (blue) and second (red) run of *L. plantarum* thermal profile.

DSC analysis confirmed that the *L. plantarum* strain showed a phase transition temperature higher than *L. acidophilus* (73.0 °C) and *L. rahmnosus* (67.7 °C), ranging from 82 °C to 119 °C with a maxima at 112 °C (Figure 1). These transformations were not found to be reversible, as no transitions were observed during cooling and subsequent reheating of the samples. Thus, among the investigated bacteria, *L. Plantarum* from Danisco was selected as it was the thermally more resistant strain [33].

**Figure 2:**
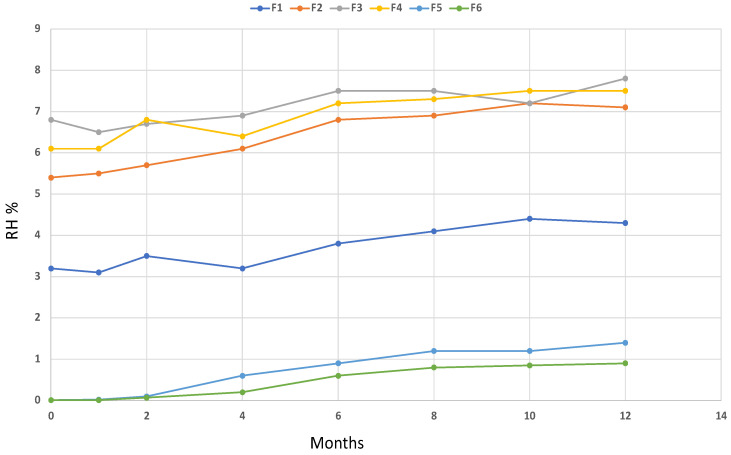
Monitoring of the RH over a period of 12 months for formulation 1 (F1, blue), formulation 2 (F2, orange), formulation 3 (F3, grey), formulation 4 (F4, yellow), formulation 5 (F5, light blue), and formulation 6 (F6, green).

Thermogravimetric analysis performed on the resulting microparticles and the calculation of the relative derivative curves allowed the calculation of the correct amount of coating with respect to the values expected in the macroparticles. The results indicate that the spray drying process under the investigated conditions led to the correct LAB/coating ratio. In addition, TGA was used to calculate the relative humidity (RH%) of the microparticles and to study its stability over a period of 12 months at room temperature. Results of the chemical characterization of the microparticles are reported in Table 1, while the monitoring of the RH over time is shown in Figure 2.

An investigation of the TG profile of the microparticles showed a correct amount of the coating material, confirming that the spray drying process was satisfactory. In addition, despite that the microparticles were prepared to be used in a nutraceutical product including fish oils, the results of the RH over 12 months showed a suitable stability over time.

### 2.3. Spray Drying Execution

A series of pilot experiments were performed to establish the process conditions of Eudragit dispersion in water with triethyl citrate (10% of the polymer) prior to spraying. The conditions monitored were the solvent systems, the feed concentrations, the flow rate, and the *L. plantarum*/polymer ratios.

A number of six feed solutions including *L. plantarum* (LP), Eudragit (Eud), and triethyl citrate were considered for spray drying experiments. In addition, the effect of different encapsulating parameters on the viability of the obtained microcapsules, such as the amount of *L. plantarum* (in LP/Eud ratios of about 1:10, 1:5, 1:2, and 1:1) and temperature (ranging from 80 °C to 110 °C) was also investigated throughout storage at room temperature.

In most studies, there was no information to support the choice of the inlet air temperature; thus, temperatures close to the solvent evaporation temperature were considered. Furthermore, most lab-scale spray-dryers, such as the one provided in this study, do not permit outlet temperature regulation, which may also affect the quality of the microcapsules. In this sense, low outlet temperatures could be considered critical, resulting in microparticles with a higher moisture content, i.e., the residual water present in the powder could act as a plasticizer, leading to physicochemical changes, such as caking, collapse, agglomeration, browning, and oxidation. In contrast, when the outlet temperature is too high, bacteria stability may be affected.

In order to increase the number of viable coated cells, in the last attempt of coating, the freeze-dried powder of *L. plantarum* was suspended in sterile water, centrifuged at 13,000 rpm for 15 min, and the products were removed from the suspension. The recovered pellet was then inserted into the feed solution. Considering the spray drying process, in all experiments the final volume of the dispersions was 100 mL, and the spraying time was 30 min.

The production yield of the spray drying process ranged from 40% to 75%.

Satisfactory process efficiency was achieved in trial 5 and trial 6, where a lower inlet temperature of 80 °C was used.

A significant decrease in yields (40% and 41%) were observed for trials 3 and 4, where the outlet temperature was lower and the extracts stuck to the wall of the inner tube in the spray drier, resulting in an incomplete evaporation of the solvent (i.e., water) which led to highly cohesive powders. The results are summarized in Table 2.

### 2.4. SEM Analysis

**Figure 3:**
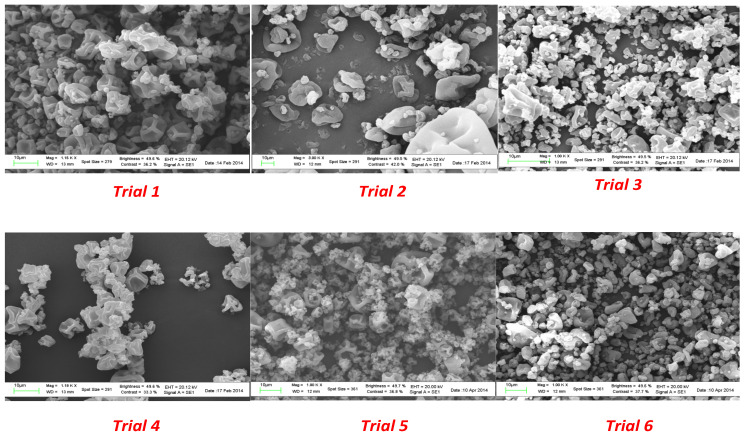
SEM images of microparticles from spray drying experiments.

Microparticles from spray drying experiments resulted in a visually uniform white powder. The size, morphology, and surface appearance were examined by scanning electron microscopy (SEM) and the obtained images are shown in Figure 3.

A surface morphology study of the powder produced in the spray drying process showed a spherical collapsed shape and a smooth surface in all the experiments. As shown in Figure 1, the use of a plasticizer provided particles that did not present breaks; in addition, the size did not exceed 400 µm. A significant reduction in the average diameter was observed as a consequence of the lower amount of coating agent from trial 1 to trial 6.

The process involves the dispersion of the core material, forming an emulsion or a dispersion, followed by homogenization of the liquid and therefore atomization of the mixture in the drying chamber leading to the evaporation of the solvent [34].

Increasing the energy provided to the atomizer decreases the size of the formed droplets. For the same energy amount, the size of formed particles increased with an increase in the feed rate (solution with the encapsulating agent and substance to encapsulate). In addition, proper adjustment and control of the working conditions, namely the inlet and outlet temperatures, led to encapsulated viable cultures with the desired particle size distribution.

**Figure 4:**
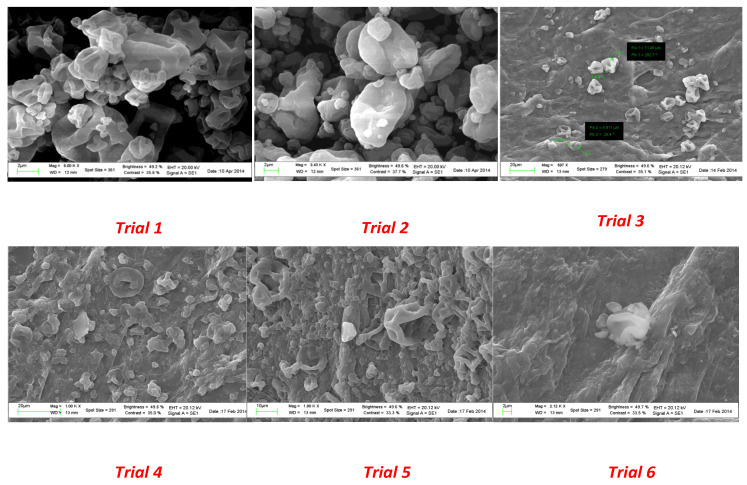
SEM images of microparticles from spray drying experiments after 2 h in an aqueous environment.

Scanning electron microscopy was also used to check the water resistance of the microparticles as well as the solubility in gastrointestinal medium. To this end, aliquots of each spray-dried powder were suspended in water for 2 h at room temperature to mimic the conditions occurring in the industrial production of capsules. As shown in Figure 4, particles from trial 1 to trial 3 revealed a higher resistance to aqueous environments. As the quantity of coating agent decreased (from trial 4 to trial 6), a partial modification of the surface morphology was observed.

**Figure 5:**
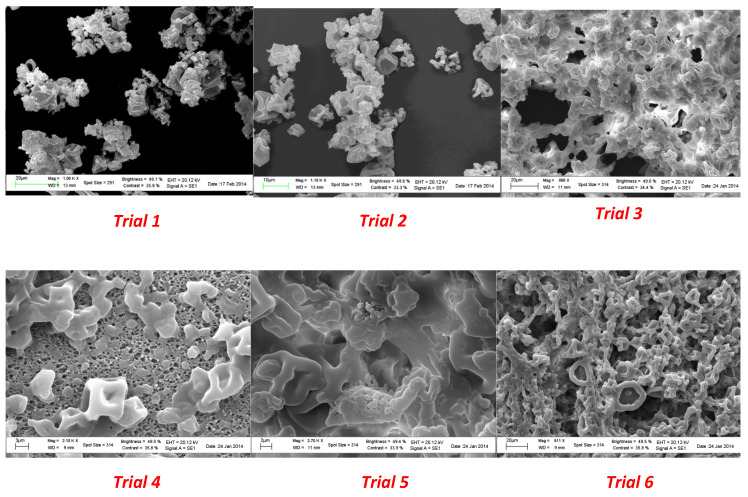
SEM images of microparticles from spray drying experiments after 2 h in HCl.

SEM images after dissolution tests revealed the absence of microorganism release in acidic medium after 2 h for all the collected powders (Figure 5); thus, microparticles will not be affected by gastric media during digestion.

**Figure 6:**
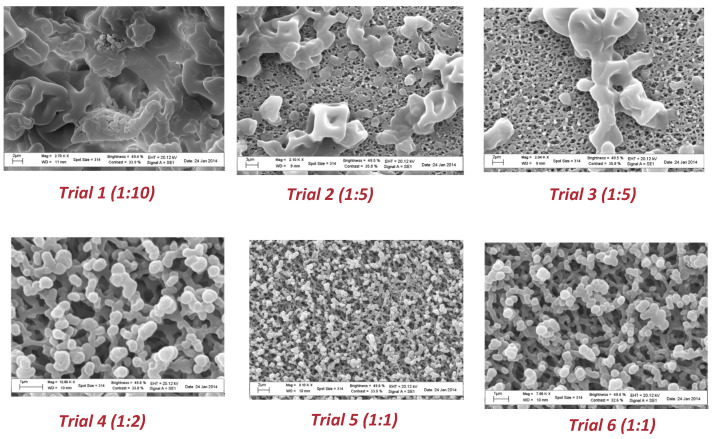
SEM images of microparticles from spray drying experiments after 24 h in PBS solution.

The same procedure was followed to verify bacteria release after incubation in PBS at pH 7.5, and the SEM images are reported in Figure 6. A good release of cell content could be observed after 24 h by spotting 10 µL of the digested medium onto a filter.

In agreement with the previous results of SEM analysis, the number of recovered lactobacilli after 24 h from trial 1 to trial 6 increased with the decreasing amount of polymer. In trials 1, 2, and 3, the bacteria release was achieved only after 48 h of incubation in PBS.

### 2.5. Enumeration of Living Cells

In order to evaluate the spray drying process efficiency with respect to viability, a series of protocols were developed for the enumeration of coated and uncoated cells. Based on the pH-dependent nature of the suggested polymer Eudragit L30 D 55, a suspension of spray-dried microcapsules in water was prepared and plated onto MRS agar plates in triplicate. The number of viable cells after dissolution in water will represent the number of viable cells not coated during the spray drying process and therefore that will be lost in the gastric media.

No growth of bacteria after incubation in water was observed for trial 1, where a ratio of 1:10 cells/polymer was used. A slightly higher growth of lactobacilli was observed in trial 3 compared to trial 2 (same percentage of cells in the feed solution) due to a lower inlet temperature of the spray. As the amount of coating agent decreased, an increasing number of uncoated cells were recovered, shown in trials 4, 5, and 6.

Meanwhile, quantity assays were performed to evaluate the gastro-resistant properties of the obtained microparticles by suspending aliquots of each powder in 0.1 M HCl and observing the absence of colony forming units in all the experiments. All the formulations showed acceptable isolation of the bacteria from the acidic medium; thus, the selection of Eudragit L30 D 55 as a hydrophobic coating material proved to be appropriate for the project requirements.

To determine the viable cell counts, the entrapped bacteria were released from the microcapsules after incubation in HCl for 2 h at 37 °C and PBS at 37 °C for 24 h, according to the protocols in 2.3.3. Quality assays for each trial were carried out to evaluate the most efficient way to achieve the complete release of the bacteria by comparing method A and method B. In Table 3, the results of the cell growth after 8 h and 24 h are summarized.

**Figure 7:**
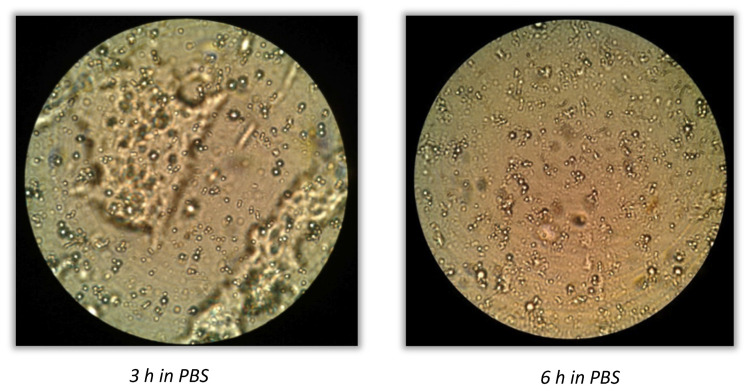
Coating removal observed by optic microscopy (100×) after 3 h and 6 h in PBS.

For method A, no colonies were observed within 8 h for microparticles from trial 1 to trial 6, the release of bacteria could be completed within 24 h for microparticles from trial 4 to trial 6, and cell growth was slightly observed after 24 h in those trials where a higher amount of coating was used. Method B resulted in a reduced time of microparticle release, allowing the coating dissolution within 8 h in almost all the formulations, concluding that this protocol led to a fast coating removal. The lower the quantity of polymer, the faster the coating removal. Complete coating removal was also observed by optical microscopy, as reported in Figure 7.

Quantity assays were performed according to method B to establish the viability of the bacteria and the results are reported in Table 4. The results of the bacteria counts showed a similar behavior between each of the different microcapsules in trial 1 to trial 5, ranging from 1.3 ± 0.5 × 102 to 1.2 ± 0.6 × 104. In contrast, the higher amount of viable cells observed in trial 6 (about 6.0 ± 0.5 × 109) is related to the increased number of starting living cells in the feed solution achieved by centrifugation and elimination of excipients.

All spray-dried microcapsules containing *L. plantarum* showed low survival rates after the spray drying process. A significant improvement in viability was observed in trial 6 compared to other trials (*p* < 0.05). Moreover, according to the recommended levels of probiotic food and taking into account the starting number of encapsulated bacteria, the count of viable probiotic cells is suitable, even in trial 6 where a higher amount of living cells were recovered. In addition, in some cases, the coating removal under simulated conditions requires too many hours to be completed; therefore, an alternative dissolution test, by means of simulated gastric fluid (SGF, containing pepsin) and simulated intestinal fluid (SIF, containing pancreatin), was considered to investigate the feasibility of the microparticles in food supplements as dietary products.

A significant improvement in bacteria release and time taken for bacteria release was achieved for all the formulations, as observed from the plates of all the trials in incubation times of 1 h to 6 h.

### 2.6. Stability Tests with Filler Solutions

Compatibility assays with selected nutrients were carried out to check the possibility of including coated probiotics in some emerging supplements.

Three filler solutions were considered in this study, including cod liver oil (Filler A), docosahexaenoic acid (Filler B), and refined docosahexaenoic acid (Filler C).

The stability of the spray-dried microparticles was tested by suspending aliquots of all the formulations in each liquid filler, and the enumeration of the colony forming units (CFU) was calculated over time in order to achieve a 12-month shelf life in non-refrigerated storage conditions. The effect of filler solutions on the viability of coated and uncoated *L. plantarum* is presented in Table 5 and Table 6.

Encapsulation of *L. plantarum* in Eudragit microspheres could significantly improve the survival of bacteria, as shown in Table 5 (*p* < 0.05). Uncoated cells completely lost their viability in filler solutions after a few days of exposure, while the results for coated bacteria clearly indicated that Eudragit microspheres could provide a significantly good protection against the damage of some nutrients commonly used in supplements. In all cases, a loss of no more than one order of magnitude (1 log CFU/g) was observed over a period of 12 months (Table 6), suggesting that the microencapsulation process may be performed by spraying a suitable suspension of selected and pretreated LAB.

Despite several studies reporting the microencapsulation of LAB with different coatings, the novelty of this work is related to the storage stability obtained; in fact, the microencapsulation process under the reported conditions resulted in bacterial survival even after prolonged and non-simulated storage [17,35,36].

## 3. Materials and Methods

### 3.1. Materials, Strains and Coatings Selection

In this study, lactic acid bacteria (LAC) such as *L. plantarum LP115* supplied from Danisco spa (Agrate Brianza, Italy) was used for the microencapsulation of LAB by spray drying technology. These strains were cultured in 30 mL of agar and MRS-broth (Difco™ BD, Sparks, MD, USA) under anaerobic conditions at 37 °C for 18 h for biomass production.

Eudragit L30 D-55 (Methacrylic Acid–Ethyl Acrylate Copolymer 1:1) was supplied from Evonik Industries (AG, Darmstadt, Germany) as a 30% aqueous dispersion soluble above pH 5.5. Triethyl citrate, supplied from Merk Millipore (Darmstadt, Germany), was used as plasticizer to be added to Eudragit dispersions.

Dissolution tests were performed with hydrocloric acid (HCl at pH 2) and phosphate buffer solution (PBS) at a pH value of 7.5 according to Pharmacopeia 8.0. Tween 80 (Sigma, Milan, Italy) was used as a non-ionic detergent for coating removal.

All other reagents were analytical grade and Milli-Q water (Millipore, Molsheim, France) was used throughout.

### 3.2. Spray Drying Execution

Freeze-dried probiotic cells were rehydrated in sterile water prior to microencapsulation by the spray drying process and stirred for 10 min at room temperature. Simultaneously, a 30% acqueous solution of Eudragit^®^ L30 D-55 containing triethyl citrate (10% *v*/*v*) was prepared and mixed to cell suspension at different ratios by stirring the mixture for 15 min until an homogeneous suspension occurred. The final volume of the suspension was 100 mL. Six feed solutions were prepared following the procedure described in order to establish the correct ratio between cells and polymer, as well as the instrument conditions needed to obtain stable microparticles with the correct number of living cells. In trial 6, the freeze-dried probiotic cells were suspended in sterile water and centrifuged for 15 min at 13,000 rpm in order to concentrate the cells in the feed solution.

These liquid feed solutions were spray-dried using a Buchi mini spray-dryer B-191 (Buchi Laboratoriums-Tecnik, Flawil, Switzerland) under the following experimental conditions: inlet temperature ranging from 80 °C to 110 °C, an outlet temperature ranging from 54 °C to 79 °C, and a feed rate of 5 mL/min. The nozzle diameter was 0.5 mm and the drying air flow was 500 l/h.

### 3.3. Thermal Analysis and Calorimetry

Differential scanning calorimetry (DSC) experiments were performed by a Perkin Elmer DSC7 calorimeter (Perkin Elmer, Boston, MA, USA) equipped with a refrigerated cooling system. The enthalpy response was calibrated using indium. The freeze-dried LAB were analyzed in DSC aluminum pans and under a continuous dry nitrogen purge (100 mL/min). Samples (10 mg) were weighed and heated over the temperature range of 20–200 °C at a heating rate of 40 °C/min in order to mimic the processes occurring in the spray drying chamber.

Thermogravimetric analysis (TGA) was used to determine the effectiveness of the coating process and the moisture of manufactured coated bacteria. Aliquots of each sample were placed into the crucible of a Perkin Elmer TGA7 Thermobalance (Massachusetts, USA), where sample temperature was measured using a thermocouple directly attached to the crucible [37,38,39,40]. The temperature was raised from room temperature (20 °C) to 800 °C with a 10 °C/min heating rate. The carrier gas (air flow) was maintained at a 100 mL/min flow rate. To ensure an accurate measurement of the sample temperature, a temperature calibration was performed using the Curie point transition of standard metals, as specified by the equipment recommendations. Each sample was analyzed in triplicate and a high reproducibility of the resulting curves was observed.

### 3.4. Scanning Electron Microscopy and Optical Microscopy

The morphology of microparticles from all the experiments was evaluated by scanning electron microscopy (SEM) using the LEO1450VP along with the program EDS, INCA300. Before observing the surface, the powders were placed on a piece of adhesive paper fixed on aluminum stubs and coated with gold with a vacuum sputtering coater. Images were acquired at 5 kV of acceleration and 1750 mA. An optical microscope provided by Carl Zeiss (Jena, Germany) was used to observe the coating removal after the dissolution tests, in order to confirm the release of the microparticles and to compare with the results from viability tests.

### 3.5. Enumeration of Living Cells

Microbiological tests were performed to establish the number of living cells after each coating process. Based on European Pharmacopeia 8.0, several procedures were carried out in order to quantify the starting viable cell count before coating, to quantify the correct number of coated and uncoated cells, and to identify the viable cells as belonging to those enclosed in the microspheres.

The enumeration of *L. plantarum* before the coating process consisted of a suspension of 10 mg of freeze-dried cells (concentration achieved: 1 mg/mL) in sterile H_2_O, stirred for 2 min to dissolve cells uniformly. Serial decimal dilutions were prepared and plated onto MRS media in triplicate before incubating the plates at 37 °C for 48 h.

The number of observed colonies on the MRS agar plates were recorded as colony forming units per gram (CFU/g); that is, viable *L. plantarum* cells per gram, taking into account the dilution factor of the plates counted. Only plates with between 25 and 450 colonies were counted.

The same procedure was followed before each coating attempt in order to evaluate the correct number of viable cells entrapped in the microparticles.

Dissolution tests were performed according to European Pharmacopeia 8.0 for conventional release of oral dosage forms, and the enumeration of coated and uncoated cells was established. With the aim of estimating the number of uncoated cells, the encapsulated probiotic bacteria were incubated in sterile water for 2 h at 37 °C, while the coated cells were evaluated by suspending bacteria in HCl at 37 °C for 2 h. Bacteria were then recovered by centrifugation at 8000× *g* for 15 min (Allegra X-22R Centrifuge, Beckman Coulter, Fullerton, CA, USA) and incubated in phosphate buffer solution (PBS) for 4 h at 37 °C (method A). The evaluation of bacterial viability was carried out by plating serial dilutions of the resulting suspension into MRS medium. The number of the released cells after acidic medium and PBS indicates the amount bacteria reaching the gut, considered as objective of this project. The cells observed after water suspensions indicate the number of uncoated bacteria that will be lost after intake, and was used to estimate the encapsulation efficiency after each coating attempt.

Finally, the performances of the alternative dissolution protocol (method B) were evaluated to determine the entrapped cell counts based on the suspension of the microparticles in HCl for 2 h at 37 °C. Instead of replacing the acid medium, the pH was adjusted to 7.5 with PBS (pH value of 11.8) for 24 h at 37 °C.

The addition of pepsin and pancreatin enzymes into the dissolution media was performed to simulate gastro-intestinal conditions, and to this end, simulated gastric and enteric fluids were prepared according to European Pharmacopeia 8.0 protocols.

All the results were presented as the mean of three counts ± standard deviation. Comparisons between the various sets of data were carried by ANOVA and *t*-tests. A *p*-value below 0.05 (presented as *p* < 0.05) was considered statistically significant.

### 3.6. Compatibility Assays

The compatibility between lactic acid bacteria (LAB) and the coating was analyzed in order to identify the correct coating material among those allowed as food ingredients. An amount of 10 mg of uncoated LAB was suspended in coating agent, and the viability in accelerated conditions was evaluated for 12 months.

The suitability of the coating material was also verified by means of a dissolution test, in order to verify the gastro resistance as well as the microparticles release, i.e., coating removal in the gut.

In addition, the survival of all the encapsulated probiotics in fish oil was confirmed in order to achieve a 12-month shelf life in non-refrigerated storage conditions. An amount of 1 g of each manufactured powder was suspended in 100 mL of the selected nutrients of interest and the enumeration of the living cells was estimated periodically; 1 mL of the suspension was centrifuged at 10,000 rpm for 10 min, the supernatant was removed, and the resulting pellet was suspended in 1 mL of sterile water prior to plating, as previously described.

## 4. Conclusions

Since the effectiveness of probiotic consumption on human health is related to their viability, it is important to minimize cell death during the microencapsulation process to ensure minimal loss of viability of the microencapsulated bacteria during storage.

The novelty of this study consists of the optimization of a microencapsulation technology based on spray drying to introduce coated probiotics into some emerging nutraceutical supplements that were able to offer a dual positive effect, i.e., the presence of lactic acid bacteria and fish oils in a single nutraceutical capsule.

Based on the preliminary achieved results, spray drying proved to be a promising technology for producing stable coated probiotics with the desired size and morphology, as confirmed by an SEM investigation. In addition, the thermal analysis characterization demonstrated that the process was satisfactory in terms of yield of production, relative humidity, and the calculated amount coating. Moreover, this technique is highly reproducible, rapid and relatively low cost; some of the most important issues for industrial application. The results showed that for almost all the probiotic-loaded microparticles, the shelf-life was extended to 12 months in non-refrigerated storage conditions.

## Figures and Tables

**Table 1:** Results of relative humidity (RH) and correct coating amount calculated by the TGA process for each coating attempt.

Trial	Cells/Eudragit Ratio	Calculated Eudragit (%)	RH (%)
1	1:10	80.7	3.2
2	1:5	75.0	5.4
3	1:5	78.3	6.8
4	1:2	61.2	6.1
5	1:1	49.9	0.01
6	1:1	45.2	0.01

**Table 2:** Results of yield for each attempt of coating.

Trial	Cells/Eudragit Ratio	Plasticizer (%)	Inlet Temperature (°C)	Outlet Temperature (°C)	Yield (%)
1	1:10	10	110	77	60.0
2	1:5	10	110	72	58.9
3	1:5	10	90	69	41.8
4	1:2	10	90	55	40.0
5	1:1	10	80	56	73.4
6	1:1	10	80	50	85.0

**Table 3:** Results of the cell growth after 8 h and 24 h according to methods A and B.

Trial	Cells:Polymer Ratio	Method A Growth (8 h)	Method A Growth (24 h)	Method B Growth (8 h)	Method B Growth (24 h)
1	1:10	Not Observed	Slightly observed	Not observed	Observed
2	1:5	Not Observed	Slightly observed	Slightly observed	Observed
3	1:5	Not Observed	Slightly observed	Slightly observed	Observed
4	1:2	Not Observed	Observed	Observed	Observed
5	1:1	Not Observed	Observed	Observed	Observed
6	1:1	Not Observed	Observed	Observed	Observed

**Table 4:** Quantity assays performed on coated *L. plantarum* according to method B, compared to uncoated cells.

Trial	LP/Eudragit Ratio	Viability in H_2_O (CFU/g)	Viability in HCl (CFU/g)	Viability in PBS (CFU/g)
1	1:10	-	Absent	2.0 ± 0.4 × 10^3^
2	1:5	1.1 ± 0.7 × 10^2^	Absent	1.2 ± 0.6 × 10^4^
3	1:5	2.1 ± 0.7 × 10^3^	Absent	1.1 ± 0.5 × 10^4^
4	1:2	5.3 ± 0.1 × 10^3^	Absent	1.3 ± 0.5 × 10^2^
5	1:1	1.1 ± 0.7 × 10^3^	Absent	8.0 ± 0.2 × 10^3^
6	1:1	2.3 ± 0.5 × 10^2^	Absent	6.0 ± 0.5 × 10^9^
*L. plantarum*		2.1 ± 0.5 × 10^11^	Absent	2.0 ± 0.3 × 10^11^

**Table 5:** Evaluation of the effect of filler solutions on the viability of coated and uncoated *L. plantarum* after 6 months.

Trial	Uncoated Reference (CFU/g)	Coated Reference (CFU/g)	Filler A (CFU/g)	Filler B (CFU/g)	Filler C (CFU/g)
1	4.0 ± 0.2 × 10^11^	2.0 ± 0.4 × 10^3^	2.5 ± 0.5 × 10^3^	1.7 ± 0.2 × 10^3^	5.1 ± 0.3 × 10^3^
2	4.0 ± 0.2 × 10^11^	1.2 ± 0.6 ×10^4^	1.1 ± 0.2 × 10^4^	0.9 ± 0.3 × 10^3^	1.0 ± 0.4 × 10^4^
3	4.0 ± 0.2 × 10^11^	1.1 ± 0.5 × 10^4^	4.6 ± 0.2 × 10^4^	2.9 ± 0.5 × 10^4^	8.2 ± 0.6 × 10^4^
4	4.0 ± 0.2 × 10^11^	1.3 ± 0.5 × 10^2^	1.6 ± 0.1 × 10^2^	1.2 ± 0.5 × 10^2^	1.0 ± 0.2 × 10^2^
5	2.0 ± 0.1 × 10^11^	8.0 ± 0.2 × 10^3^	1.4 ± 0.6 × 10^3^	2.7 ± 0.7 × 10^2^	3.8 ± 0.2 × 10^3^
6	2.1 ± 0.4 × 10^11^	6.0 ± 0.5 × 10^9^	2.9 ± 0.4 × 10^9^	4.4 ± 0.9 × 10^8^	1.0 ± 0.3 × 10^8^

**Table 6:** Evaluation of the effect of filler solutions on the viability of coated and uncoated *L. plantarum* after 12 months.

Trial	Uncoated Reference (CFU/g)	Coated Reference (CFU/g)	Filler A (CFU/g)	Filler B (CFU/g)	Filler C (CFU/g)
1	4.0 ± 0.2 × 10^11^	2.0 ± 0.4 × 10^3^	1.5 ± 0.5 × 10^2^	-	1.2 ± 0.3 × 10^2^
2	4.0 ± 0.2 × 10^11^	1.2 ± 0.6 ×10^4^	1.2 ± 0.2 × 10^2^	-	2.0 ± 0.4 × 10^2^
3	4.0 ± 0.2 × 10^11^	1.1 ± 0.5 × 10^4^	2.4 ± 0.1 × 10^3^	-	1.3 ± 0.1 × 10^3^
4	4.0 ± 0.2 × 10^11^	1.3 ± 0.5 × 10^2^	1.1 ± 0.2 × 10^2^	1.3 ± 0.1 × 10^2^	1.0 ± 0.1 × 10^2^
5	2.0 ± 0.1 × 10^11^	8.0 ± 0.2 × 10^3^	1.1 ± 0.5 × 10^2^	-	1.2 ± 0.3 × 10^2^
6	2.1 ± 0.4 × 10^11^	6.0 ± 0.5 × 10^9^	2.1 ± 0.2 × 10^9^	2.3 ± 0.4 × 10^8^	1.8 ± 0.2 × 10^8^
